# Supplementary material for: Mapping pesticide mixtures to cancer risk at the country scale with spatial exposomics
Source: Nat Health. 2026 Apr 1;1(5):520–31. doi: 10.1038/s44360-026-00087-0 (PMC13156043; doi:10.1038/s44360-026-00087-0)
Supplement: Supplementary file 1 — Supplementary Methods, Tables 1–3, Figs. 1–4 and References. [file 44360_2026_87_MOESM1_ESM.pdf]

nature health

Supplementary information https://doi.org/10.1038/s44360-026-00087-0

Mapping pesticide mixtures to cancer risk at the country scale with spatial exposomics

In the format provided by the authors and unedited

Supplementary Information Guide

Supplementary Methods.. 2
Supplementary Fig. 1 6
Supplementary Fig. 2 7
Supplementary Fig. 3 8
Supplementary Fig. 4 9
Supplementary Table 1 10
Supplementary Table 2 11
Supplementary Table 3 12
Supplementary References 13

Supplementary Methods

1. Correction factor f

The correction factor f, applied to the expression shown in Fig. 1 to account for terrain slope, plant interception and the presence of buffer zones, was calculated as follows:

$$f = f_1 \times f_2 \times f_3$$

- f_1: factor reflecting the influence of the slope on L%
  - if slope < 20%: $f_1 = 0.02153 \times \text{slope} + 0.001423 \times \text{slope}^2$
  - if slope ≥ 20%: $f_1 = 1$

adapted from Beinat & van den Berg, 1996 (ref. 78)

- f_2: factor reflecting the influence of pesticide intercepted by plant cover on L%
  $f_2 = 1 - PI / 100$
  PI = plant interception (%)
  A value of PI = 0 was assumed.

- f_3: factor reflecting the mitigating effect of a densely vegetated buffer zone on L%
  $f_3 = 0.83^{WBZ}$
  WBZ = width of the buffer zone (m).
  If no densely vegetated buffer is present, WBZ was set to zero.

2. Estimation of K_{oc}

In cases where experimental values were unavailable, K_{oc} was estimated as:

$$\log K_{oc} = 1.029 \times \log K_{ow} - 0.18$$

from Rao & Davidson, 1980 (ref. 79)

3. Multi-residue chemical analyses

Levels of 67 AIs and degradation products—spanning 19 pesticides incorporated in the environmental exposure risk model—were quantified in hair samples (pg mg^{-1}) using liquid chromatography with tandem mass spectrometry (LC–MS/MS) or gas chromatography with tandem mass spectrometry (GC–MS/MS) (see Supplementary Table 1)[19].

A 3-cm section from the hair root was cut into small pieces and pulverised at 25 Hz for 2 min in a Mixer Mill MM400 (Retsch), and 50 mg of hair powder were suspended in 1 mL of a 50:50 (v/v) acetonitrile (ACN)/methanol (MeOH) mixture. Next, 50 µL of a deuterated internal standard stock solution, consisting of p,p'-dichlorodiphenyltrichloroethane (DDT)-d8 (CAS: 93952-18-2; 5 µg mL^{-1}), acetochlor-d11 (CAS: 1189897-44-6; 5 µg mL^{-1}), atrazine-d5 (CAS: 163165-75-1; 5 µg mL^{-1}), isoproturon-d6 (CAS: 217487-17-7; 5 µg mL^{-1}) and simazine-d10

(CAS: 220621-39-6; 1 µg mL^{-1}) in a 50:50 ACN/MeOH mixture, was added. The suspension was sonicated at room temperature for 15 min using an Ultrasonic Cleaner (VWR), then centrifuged for 5 min at 4,000 × *g* before collecting the supernatant for pesticide detection and quantification.

LC–MS/MS samples were analysed using a 1290 Infinity Liquid Chromatography System coupled to a 6460 Triple Quadrupole Mass Spectrometer with a G4226A Autosampler (Agilent Technologies). Chromatographic separation was achieved on a Nucleodur C18 HTec column (100 × 2 mm I.D., 1.8 µm; Macherey–Nagel) using a mobile phase comprising water with 0.1% formic acid (A) and ACN (B) at a flow rate of 0.7 mL min^{-1}. The gradient profile was as follows: isocratic elution with 95% A and 5% B for 1 min; a gradient from 5% to 73% B over 11 min; and a linear gradient from 73% to 90% B over 30 s, followed by re-equilibration for 1.5 min to restore initial conditions. The column was maintained at 50 °C, the autosampler at 5 °C and the injection volume was 5 µL. The mass spectrometer, equipped with either a Jet Stream electrospray ionisation (ESI) source or an atmospheric pressure chemical ionisation (APCI) source (Agilent Technologies), depending on the analyte, operated in multiple reaction monitoring (MRM) mode under both positive and negative ionisation conditions. The nebulisation gas was heated to 300 °C (5 L min^{-1}, 45 psi), and the sheath gas to 300 °C (10 L min^{-1}), with an ionisation spray voltage of 3.5 kV. Two or three main transitions per standard were monitored, and calibration curves were generated using standard concentrations ranging from 0.2 to 200 ng mL^{-1} in a 50:50 ACN/MeOH mixture.

GC–MS/MS samples were analysed using a 7890A Gas Chromatograph equipped with a multimode inlet and coupled to a 7000B Triple Quadrupole Mass Spectrometer (Agilent Technologies) with a MultiPurposeSampler MPS Robotic Pro autosampler (Gerstel) and an HP-5 ms Ultra Inert column (30 m × 0.25 mm I.D., 0.25 µm; Agilent Technologies). The oven programme consisted of an initial hold at 60 °C for 2 min, followed by a ramp of 60 °C min^{-1} to 180 °C, a ramp of 5 °C min^{-1} to 240 °C, a ramp of 60 °C min^{-1} to 300 °C and a final hold at 300 °C for 4 min. The injector, fitted with a Gooseneck Splitless Liner (4 mm I.D.; Restek), was maintained at 300 °C. Injections of 4 µL were performed in splitless mode, with helium as the carrier gas at 1 mL min^{-1}, and the transfer line was set to 300 °C. The mass spectrometer, fitted with an inert electron impact (EI) source (G7000-65710; Agilent Technologies) operating in MRM mode, utilised helium and nitrogen (purity ≥ 99.9999%) as collision gases, with the source maintained at 230 °C. Two or three main transitions per standard were monitored, and calibration curves were constructed using standard concentrations ranging from 0.2 to 200 ng mL^{-1} in a 50:50 ACN/MeOH mixture.

Calibration curves were generated using a blank hair matrix confirmed to be free or below limit of detection (LOD), sourced from a hairdresser in Strasbourg (France) covering 10 measurement points ranging from 4 to 4,000 pg mg^{-1}. Randomized samples spiked with deuterated internal standards were analysed on both LC–MS/MS and GC–MS/MS systems,

with calibration checks performed every ten samples during each batch run. Analytes were identified by their retention times and the ratio of qualifier to quantifier signals, and quantification was achieved using calibration curves derived from adjacent calibration checks. All analytical standards were purchased from Sigma-Aldrich and LGC. ACN and MeOH, both high-performance liquid chromatography (HPLC) grade, were obtained from Carlo Erba Reagents.

A hair sample was deemed contaminated if its contaminant concentration exceeded the LOD. For samples with concentrations between the LOD and the limit of quantification (LOQ)—that is, below the concentration of the lowest calibrator—the estimated concentration was calculated using a value of LOQ/√2 (ref. 80).

4. Histopathological assessment of liver tissue

HCC and paired NTL samples were obtained as detailed in the Methods. Tissues were formalin-fixed and paraffin-embedded (FFPE) at the INEN Department of Pathology using standard protocols[42]. HCC diagnosis followed the WHO classification[81]. Hepatic steatosis was graded (0–3) on haematoxylin–eosin-stained FFPE NTL sections at 40× magnification, according to the criteria of the Non-alcoholic Steatohepatitis Clinical Research Network (NASH CRN)[82]. Steatohepatitis was diagnosed following the guidelines of the American Association for the Study of Liver Diseases (AASLD)[83]. Fibrosis stage (F0–F4) was performed on Masson's trichrome-stained FFPE sections (Artisan kit, Agilent Dako) using the Scheuer scoring system[84].

5. Detection of aflatoxin B_1-induced hotspot mutation

HCC and paired NTL tissues were pulverised under liquid nitrogen and digested for 8 h at 37 °C in lysis buffer containing SDS and proteinase K. Total DNA was extracted twice with phenol and once with chloroform, precipitated with ethanol and resuspended in TE buffer (10 mM Tris, 0.1 mM EDTA, pH 8.0). DNA concentrations were measured using the Qubit dsDNA Broad-Range Assay Kit (Invitrogen). The AFB_1-induced R249S hotspot mutation in the *TP53* gene[85,86], which encodes the tumour protein p53, was assessed by targeted PCR amplification and sequencing of exon 7 using the BigDye Terminator v3.1 Cycle Sequencing Kit (Applied Biosystems). All samples were sequenced on both strands. For each individual, HCC and matched NTL DNA were analysed in parallel to determine the somatic status of any variant detected. PCR amplifications were performed using the following primers: forward 5'-GCGCACTGGCCTCATCTTGG-3' and reverse 5'-TGGGAGCAGTAAGGAGATTC-3' (302 bp; chr17:7674027–7674328, GRCh38/hg38; https://genome.ucsc.edu/cgi-bin/hgPcr)[87].

6. Mitochondrial haplogroup determination

Mitochondrial DNA was obtained from the same HCC and NTL DNA extracts described above and used to amplify the D-loop hypervariable region 1 (HVR1), which harbours informative variants for Native American haplogroup assignment[88]. Amplification was performed using primers mt16023 (5'-GTTCTTTCATGGGGAAGCA-3') and mt16422 (5'-

ATTGATTTCACGGAGGATGG-3')[18]. Each 50 µL PCR reaction contained 10 ng of DNA, 2 U Platinum Taq DNA polymerase (Invitrogen) and 20 pmol of each primer. Touchdown PCR began with 94 °C for 2 min, followed by 18 cycles of 94 °C for 15 s, annealing from 70 °C (decreasing by 3 °C every three cycles) for 15 s and extension at 72 °C for 30 s; then 20 additional cycles of 94 °C for 15 s, 50 °C for 15 s and 72 °C for 30 s; followed by a final extension at 72 °C for 3 min. PCR products were sequenced by Sanger methodology, and haplotypes were assigned using the Mitomaster sequence analysis tool (v.Beta 1)[89].

7. Detection of hepatitis B virus

Hepatitis B surface antigen (HBsAg) status was determined serologically using the Elecsys HBsAg II assay (Roche Diagnostics), according to the manufacturer's protocol. Intrahepatic HBV DNA was quantified from the same HCC and NTL DNA extracts described above, following established protocols[44]. Digital droplet PCR (ddPCR) was performed on a QX100 Droplet Digital PCR System (Bio-Rad) using the TaqMan Pathogen Detection Assay Pa03453406_s1 and the Human TaqMan Copy Number Reference Assay (both Thermo Fisher Scientific) as a nuclear DNA reference. Each 20 µL reaction comprised 10 µL ddPCR Supermix (Bio-Rad), 1× primer–probe mix and 100 ng of total DNA. Droplets were generated with 70 µL of Droplet Generation Oil (Bio-Rad) on a QX100 Droplet Generator and transferred to a 96-well PCR plate. Duplex PCRs were run on a CM1000 Touch Thermal Cycler (Bio-Rad) with the following thermal cycling protocol: 95 °C for 10 min; 40 cycles at 94 °C for 30 s (ramp rate 2.5 °C s^{-1}) and 59 °C for 1 min (ramp rate 2.5 °C s^{-1}); followed by 98 °C for 5 min. PCR products were read on a QX100 Droplet Reader (Bio-Rad), and data were analysed using QuantaSoft software (Bio-Rad) with autoanalysis settings for duplex experiments.

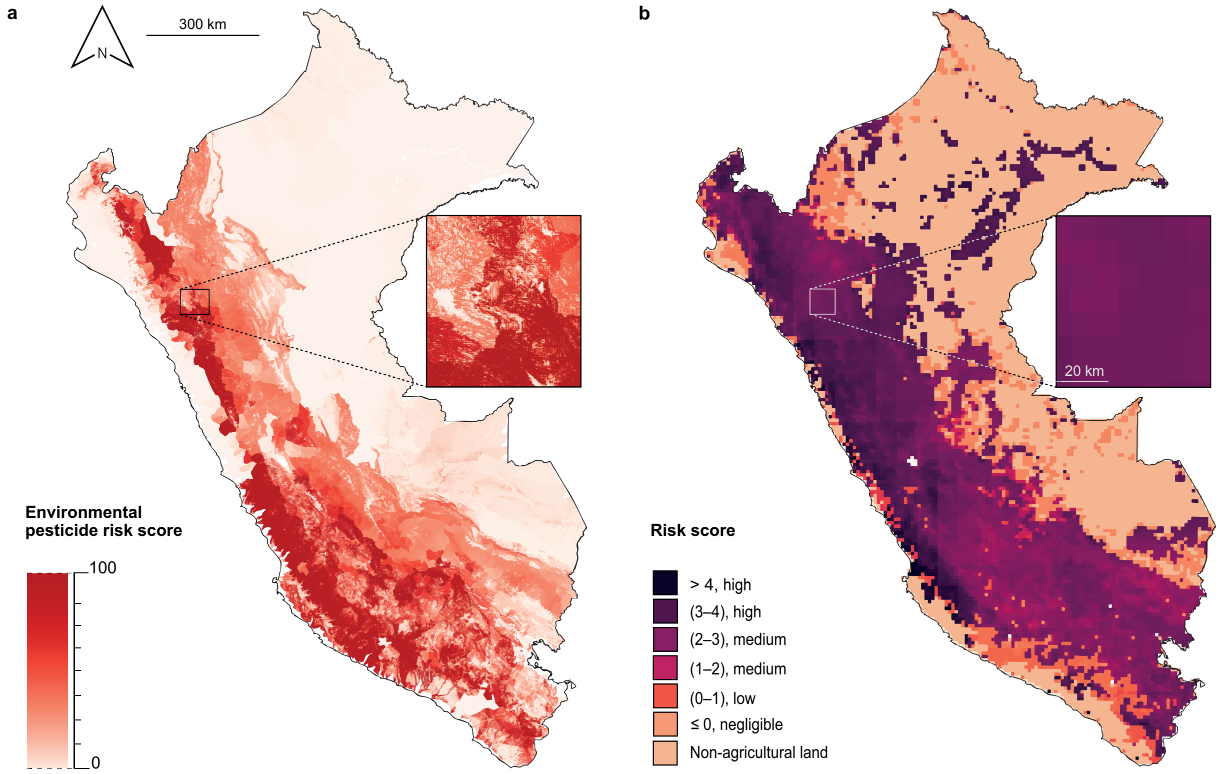

Supplementary Fig. 1 | Comparative spatial resolution of the national environmental pesticide exposure risk model and the current global reference in Peru. Environmental pesticide risk across Peru is mapped using (**a**) the national model developed in the present work, at 3.2 arcsec resolution at the equator (100 m × 100 m), as in Fig. 1; and (**b**) the model by Tang *et al.*[20], derived from a global georeferenced dataset (PEST-CHEMGRIDS v.1.01; https://sites.google.com/site/thebrtsimproject/home)[90], gridded at 5 arcmin resolution at the equator (≈10 km × 10 km). Insets show the same high-risk region under both models, illustrating the enhanced spatial granularity achieved through national-scale modelling.

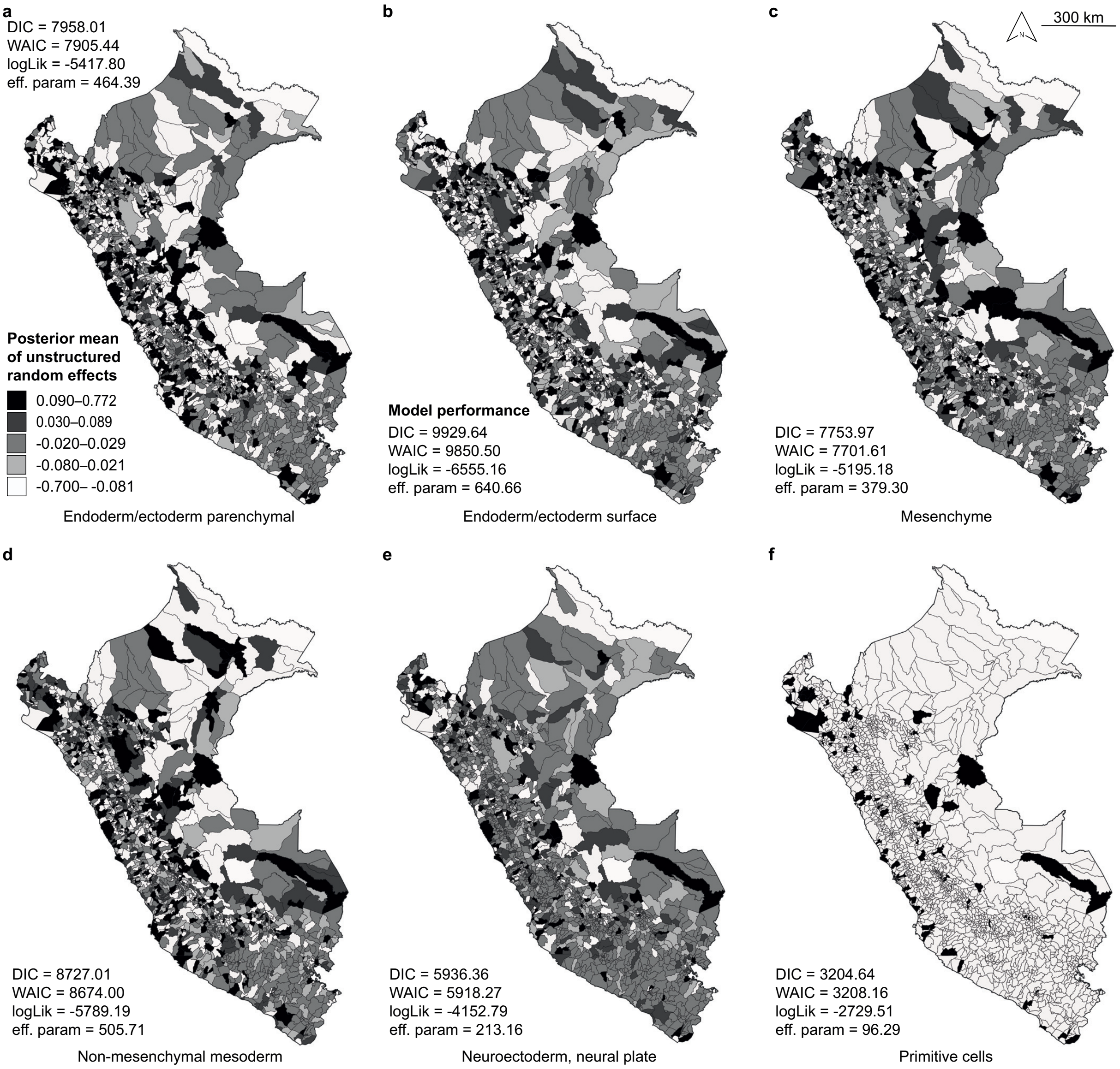

Supplementary Fig. 2 | Unstructured random effects and model performance across developmental lineages in INLA modelling. Maps in **a–f** show the spatial distributions of the posterior means of the unstructured random effects across developmental lineage categories of cancer, as in Extended Data Fig. 6, capturing residual variation not explained by fixed or spatially structured effects. Model performance is summarized by DIC, WAIC, marginal log-likelihood (logLik) and the effective number of parameters (eff. param.). Posterior estimates exhibited narrow CIs, supporting inference stability. **a,** endoderm/ectoderm-derived parenchyma; **b,** endoderm/ectoderm-derived surface; **c,** mesenchyme; **d,** non-mesenchymal mesoderm; **e,** neuroectoderm (neural plate); **f,** primitive cells, which yielded the most parsimonious model owing to the limited number of cancer cases.

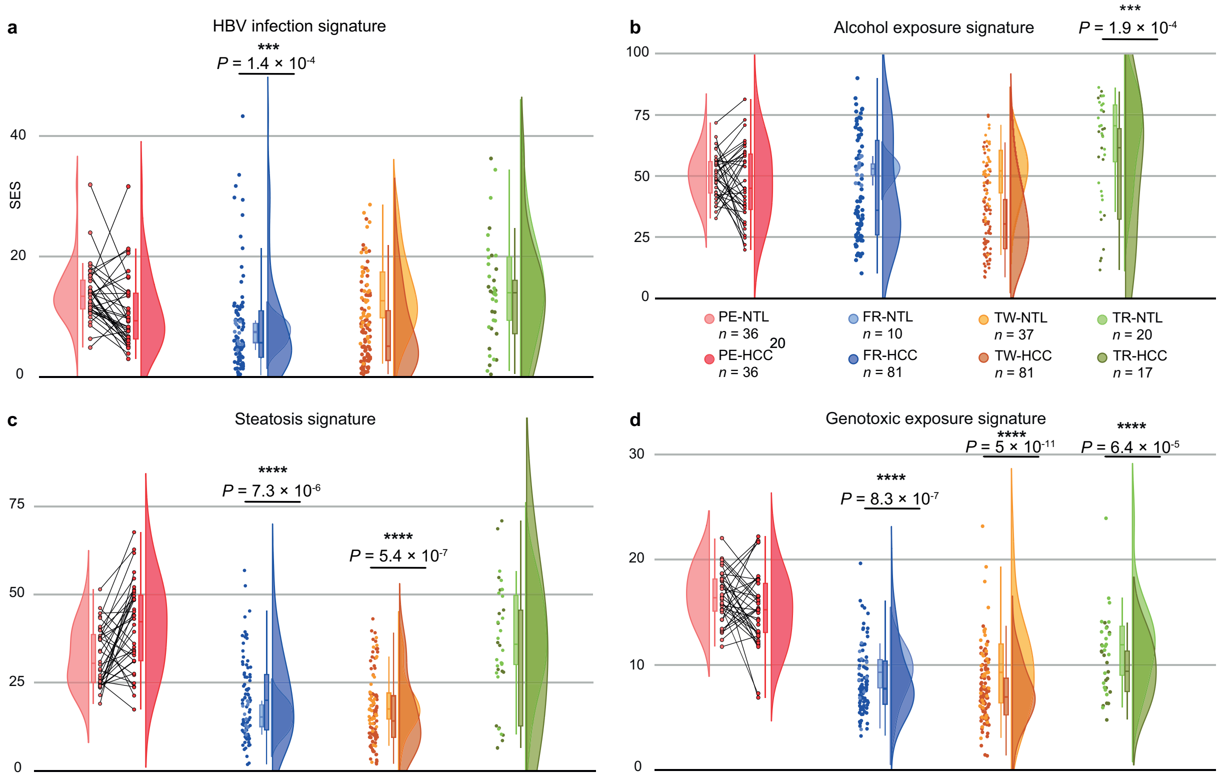

Supplementary Fig. 3 | Transcriptomic signature scores for major liver cancer risk factors across Peruvian and international cohorts. a–d, Raincloud plots of SES for curated transcriptomic signatures associated with key HCC risk factors: (**a**) HBV, (**b**) alcohol, (**c**) steatosis and (**d**) foodborne genotoxins (Extended Data Table 2). Raincloud plots combine box plots with kernel density estimates; box plots indicate the median (centre line), IQR (box) and 1.5 × IQR bounds (whiskers), with outliers beyond the whiskers plotted individually. Peruvian samples (PE; red; $n = 36$ NTL and $n = 36$ HCC) are pairwise matched and compared with cohorts from France (FR; blue; $n = 10$ NTL and $n = 81$ HCC), Taiwan (TW; orange–brown; $n = 37$ NTL and $n = 81$ HCC) and Turkey (TR; green; $n = 20$ NTL and $n = 17$ HCC); lighter and darker shades denote NTL and HCC, respectively. P values are from Kruskal–Wallis tests followed by Dunn's post hoc test with Bonferroni correction. *** $P < 0.001$; **** $P < 0.0001$ vs. Peruvian NTL samples. **d,** Peruvian NTL samples showed significantly higher SES for the genotoxic exposure signature than international cohorts ($P < 0.0001$), despite the absence of *TP53* R249S mutations—the canonical marker of AFB_1 exposure[85,86]—indicating a distinct genotoxic profile, as previously reported[15] (see Supplementary Table 2). This profile is more consistent with cumulative effects of non-genotoxic pesticide mixtures acting through synergistic mechanisms at no-observed-adverse-effect levels[46,91] (Friedman test; $Q = 112.6$, $P = 2 \times 10^{-23}$; see also Fig. 3a,b).

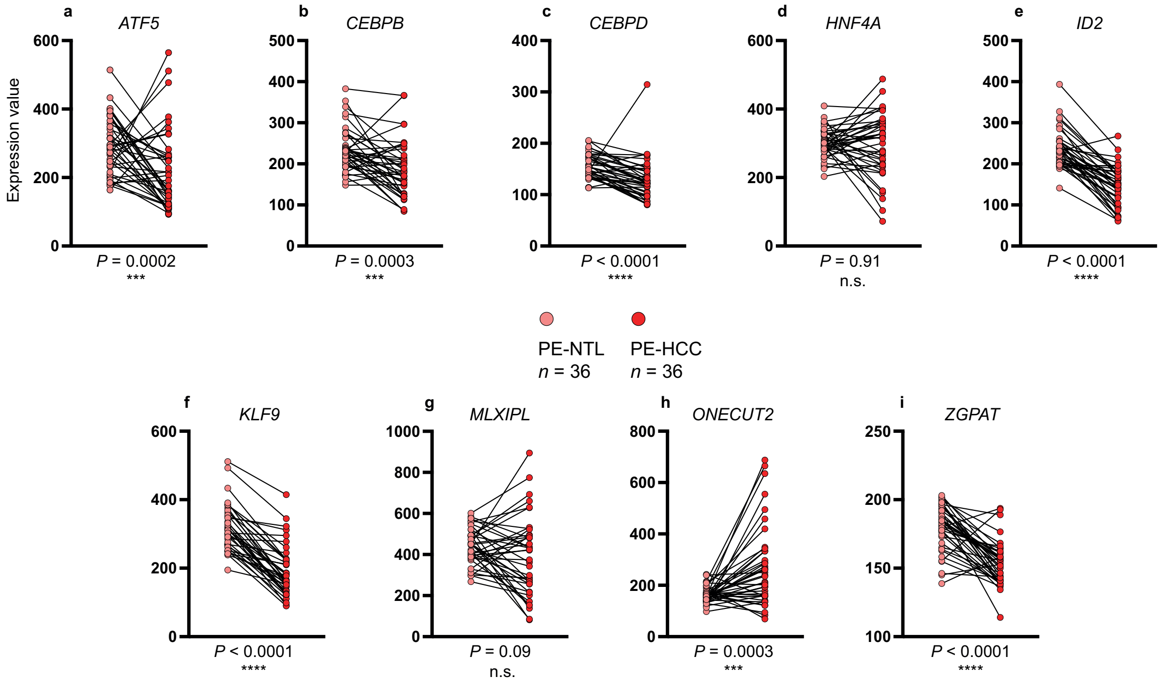

Supplementary Fig. 4 | Expression of nine lineage-specific MTFs implicated in endoderm-derived hepatobiliary carcinogenesis. a–i, Column scatterplots showing linear-scale gene expression levels of (**a**) *ATF5*, (**b**) *CEBPB*, (**c**) *CEBPD*, (**d**) *HNF4A*, (**e**) *ID2*, (**f**) *KLF9*, (**g**) *MLXIPL*, (**h**) *ONECUT2* and (**i**) *ZGPAT*—together comprising the MTF gene set (Extended Data Table 2)[26]—in Peruvian NTL (light red, $n = 36$) and matched HCC (dark red, $n = 36$) samples, as measured by gene expression microarray (GeneChip Human Transcriptome Array 2.0)[18]. *** $P < 0.001$; **** $P < 0.0001$; n.s., not significant.

Supplementary Table 1 | Analytical details of the 67 compounds quantified in hair

Compound name	CAS registry number	Substance type	Quantification method	Regulation in Peru	Included in the risk model
2,4-D	94-75-7	AI	LC–MS/MS		Yes
3-Phenoxybenzoic acid	3739-38-6	Der. synthetic pyrethroids	LC–MS/MS		Yes
4-Nitrophenol	100-02-7	Der. parathion	LC–MS/MS	Banned since 2000	
Acephate	30560-19-1	AI	LC–MS/MS		Yes
Acetamiprid	135410-20-7	AI	LC–MS/MS		
Aclonifen	74070-46-5	AI	GC–MS/MS		
Alachlor	15972-60-8	AI	GC–MS/MS		
Atrazine	1912-24-9	AI	LC–MS/MS		Yes
Azoxystrobin	131860-33-8	AI	LC–MS/MS		
Boscalid	188425-85-6	AI	LC–MS/MS		
Chlorfenvinphos	470-90-6	AI	LC–MS/MS		
CPS	2921-88-2	AI	GC–MS/MS	Banned since 2023	Yes
Cyfluthrin	68359-37-5	AI	GC–MS/MS		
Cypermethrin	52315-07-8	AI	GC–MS/MS		
Cyprodinil	121552-61-2	AI	LC–MS/MS		
DDT, p,p'-	50-29-3	AI	LC–MS/MS	Banned since 1991	
Deethylatrazine	6190-65-4	Der. atrazine	LC–MS/MS		Yes
Deltamethrin	52918-63-5	AI	GC–MS/MS		Yes
Deisopropylatrazine	1007-28-9	Der. atrazine	LC–MS/MS		Yes
DETP	2465-65-8	Der. parathion	LC–MS/MS		
Diazinon	333-41-5	AI	LC–MS/MS		Yes
Dicofol	115-32-2	AI	GC–MS/MS	Banned since 2021	
Diethyl phosphate	598-02-7	Der. diazinon	LC–MS/MS		Yes
Difenoconazole	119446-68-3	AI	LC–MS/MS		Yes
Dimethoate	60-51-5	AI	LC–MS/MS		Yes
Dimethomorph	110488-70-5	AI	LC–MS/MS		
Diuron	330-54-1	AI	LC–MS/MS		
Epoxiconazole	133855-98-8	AI	LC–MS/MS		
Fenhexamid	126833-17-8	AI	LC–MS/MS		
Fipronil	120068-37-3	AI	LC–MS/MS		Yes
Fipronil sulfone	120068-36-2	Der. fipronil	LC–MS/MS		Yes
IMHP	2814-20-2	Der. diazinon	LC–MS/MS		Yes
Imidacloprid	138261-41-3	AI	LC–MS/MS		Yes
Lindane	58-89-9	AI	GC–MS/MS	Banned since 1991	
Linuron	330-55-2	AI	LC–MS/MS		Yes
Malathion	121-75-5	AI	LC–MS/MS		
Metalaxyl-M	70630-17-0	AI	LC–MS/MS		Yes
Methamidophos	10265-92-6	AI	LC–MS/MS	Banned since 2020	Yes
Methomyl	16752-77-5	AI	LC–MS/MS		Yes
Metribuzin	21087-64-9	AI	GC–MS/MS		
Monocrotophos	6923-22-4	AI	LC–MS/MS	Banned since 2004	Yes
Myclobutanil	88671-89-0	AI	LC–MS/MS		
Oxyfluorfen	2874-03-3	AI	GC–MS/MS		Yes
Pendimethalin	40487-42-1	AI	LC–MS/MS		
Pentachloroanisole	1825-21-4	Der. pentachlorophenol	GC–MS/MS	Banned since 1999	
Piperonyl butoxide	51-03-6	Synergist agent	GC–MS/MS		
Permethrin	52645-53-1	AI	GC–MS/MS		Yes
Pirimiphos-methyl	29232-93-7	AI	GC–MS/MS		
Procymidone	32809-16-8	AI	GC–MS/MS		
Profenofos	41198-08-7	AI	LC–MS/MS		
Propiconazole	60207-90-1	AI	LC–MS/MS		
Propoxur	114-26-1	AI	LC–MS/MS		
Propylene thiourea	2122-19-2	Der. propineb	LC–MS/MS		Yes
Pyraclostrobin	175013-18-0	AI	LC–MS/MS		
Pyrimethanil	53112-28-0	AI	LC–MS/MS		
Quinoxyfen	124495-18-7	AI	LC–MS/MS		
S 421	127-90-2	Synergist agent	GC–MS/MS		
Simazine	122-34-9	AI	LC–MS/MS		
Spiroxamine	118134-30-8	AI	LC–MS/MS		
Tebuconazole	107534-96-3	AI	LC–MS/MS		Yes
Tebufenozide	112410-23-8	AI	LC–MS/MS		
Terbuthylazine	5915-41-3	AI	LC–MS/MS		
Tetramethrin	7696-12-0	AI	GC–MS/MS		
Transfluthrin	118712-89-3	AI	GC–MS/MS		
Triadimenol	55219-65-3	Der. triadimefon	LC–MS/MS		
Trifloxystrobin	141517-21-7	AI	LC–MS/MS		
λ-cyhalothrin	91465-08-6	AI	GC–MS/MS		

Abbreviations: Der, derivative of; DETP, O,O-diethyl hydrogen thiophosphate; IMHP, 2-isopropyl-6-methyl-4-pyrimidinol.

Supplementary Table 2 | Baseline characteristics of HCC patients, including demographic data, hotspot residence, mitochondrial haplotype, liver pathology and HBV markers

Patient code	Sex	Age	UBIGEO	Mitochondrial haplotype	Steatosis grade	Steatohepatitis	Fibrosis stage	*TP53* R249S mutation	HBsAg	HBV DNA (copies/cell)
PE0004	M	70	050101	B	0	Absent	1	Absent	Negative	0.001
PE0011	M	17	050101	B	0	Absent	2	Absent	Positive	0.901
PE0013	M	28	150118	B	0	Absent	0	Absent	Positive	0.730
PE0015	F	32	050101	B	0	Absent	0	Absent	Positive	0.395
PE0016	F	74	040101	B	0	Absent	0	Absent	Negative	0.002
PE0025	M	15	030415	B	0	Absent	0	Absent	Positive	0.613
PE0026	M	36	120301	B	0	Absent	1	Absent	Positive	0.005
PE0034	M	50	150125	A	0	Absent	1	Absent	Negative	0
PE0047	F	79	150143	C	0	Absent	1	Absent	Negative	0.006
PE0054	F	45	120301	D	1	Absent	1	Absent	Negative	0.310
PE0068	M	62	040302	C	0	Absent	3	Absent	Positive	0.730
PE0070	M	64	200504	B	1	Absent	1	Absent	Negative	0
PE0083	M	73	150714	B	1	Present	0	Absent	Negative	0
PE0088	M	67	150709	B	0	Absent	1	Absent	Negative	0.015
PE0090	M	27	050101	A	0	Absent	2	Absent	Negative	0.585
PE0092	M	76	120301	D	0	Absent	0	Absent	Negative	0
PE0093	M	32	200602	C	0	Absent	0	Absent	Negative	0
PE0095	M	30	050101	A	0	Absent	0	Absent	Positive	0.597
PE0099	F	36	030415	D	0	Absent	1	Absent	Positive	0.669
PE0100	F	43	100101	B	0	Absent	0	Absent	Negative	0.006
PE0101	M	23	140101	C	0	Absent	1	Absent	Positive	0.479
PE0106	F	71	090612	D	1	Absent	3	Absent	Negative	0.002
PE0109	F	36	190112	B	0	Absent	0	Absent	Negative	0
PE0116	M	77	150110	B	0	Absent	1	Absent	Negative	0
PE0117	F	43	030415	B	0	Absent	0	Absent	Negative	0
PE0120	F	19	021101	B	0	Absent	1	Absent	Positive	0.643
PE0121	F	73	120301	B	2	Present	3	Absent	Negative	0
PE0173	M	50	120301	B	0	Absent	3	Absent	Negative	0
PE0179	M	33	030415	C	1	Absent	1	Absent	Positive	0.335
PE0180	M	34	120301	B	0	Absent	0	Absent	Positive	0.205
PE0188	F	94	021101	B	0	Absent	0	Absent	Negative	0.002
PE0190	F	45	120201	A	1	Absent	1	Absent	Positive	0.178
PE0191	M	67	120301	A	0	Absent	2	Absent	Negative	0
PE0192	M	33	050101	B	3	Absent	0	Absent	Positive	0.208
PE0193	F	13	120301	A	0	Absent	0	Absent	Negative	0
PE0200	M	42	050101	D	1	Absent	2	Absent	Positive	0.764

Histological and molecular assessments are presented for NTL samples.

Supplementary Table 3 | Public databases used in the study

Name	Database	Version	Source
90m Digital Elevation Model	EarthEnv-DEM90	1	https://www.earthenv.org/DEM
Censos Nacionales 2007	National_census	2007	http://censos1.inei.gob.pe/Censos2007/redatam/
Censos Nacionales 2017	National_census	2017	https://censos2017.inei.gob.pe/pubinei/index.asp
Chemical Entities of Biological Interest	CEBI		https://www.ebi.ac.uk/chebi/
Código de Ubicación Geográfica (geographic location codes)	UBIGEO	2.0	https://webapp.inei.gob.pe:8443/sisconcode/main.htm#
Datos Espaciales del Perú (INEI shapefiles)	Cartographic layers	2023	https://ide.inei.gob.pe/#capas
Developmental Lineage Classification and Taxonomy of Neoplasms	12885_2003_100_MOESM1_ESM		https://bmccancer.biomedcentral.com/articles/10.1186/1471-2407-4-10
Global Soil Organic Carbon Map	GSOCmap-FAO		https://data.apps.fao.org/glosis/?lang=en
Harmonized World Soil Database v.1.2	T_REF_BULK	1.0	https://www.fao.org/soils-portal/soil-survey/soil-maps-and-databases/harmonized-world-soil-database-v12/en/
IARC Monographs	Classifications	v.1–139	https://monographs.iarc.who.int/list-of-classifications
Infraestructura de Datos Espaciales del Perú (IDEP)	Department, District	2021	https://www.idep.gob.pe/geovisor/descarga/visor.html
IV Censo Nacional Agropecuario 2012	National_census	v.1.0	http://censos1.inei.gob.pe/Cenagro/redatam/
NCI Thesaurus (NCIt)	NCIt_Maps_To_ICD10CM	25.03e	https://evsexplore.semantics.cancer.gov/evsexplore/welcome
Peruvian Interpolated data of SENAMHI's Climatological Observations	PISCO_HyM_GR2M	1.1	https://figshare.com/articles/dataset/PISCO_HyM_GR2M_v1_1/14382758
Pesticide Properties Database	PPD		https://sitem.herts.ac.uk/aeru/ppdb/en/
PubChem			https://pubchem.ncbi.nlm.nih.gov/
Sea Surface Temperature (SST)	OISST.v2.1, monthly	1991–20	https://www.cpc.ncep.noaa.gov/data/indices/
Servicio Nacional de Sanidad Agraria (SENASA)	SIGIA	2020	https://servicios.senasa.gob.pe/SIGIAWeb/sigia_consulta_empresa.html
Superficie Agrícola de Perú	Vectorial_map	2018	https://siea.midagri.gob.pe/portal/informativos/superficie-agricola-peruana
WRI Global Forest Watch	Emerging hot spots	2002–23	https://www.globalforestwatch.org
X-GRIDS BRTSim project	PEST-CHEMGRIDS	v.1.01	https://sites.google.com/site/thebrtsimproject/x-grids-downloads

Supplementary References

78. Beinat, E. & van den Berg, R. *EUPHIDS, a Decision Support System for the Admission of Pesticides*. 196 (Dutch National Institute of Public Health and the Environment, 1996).
79. Rao, P. S. C. & Davidson, J. M. Estimation of pesticide retention and transformation parameters required in non-point source pollution models. in *Environmental Impact of Non-Point Source Pollution* (eds Overcash, M. R. & Davidson, J. M.) 23–67 (Ann Arbor Science Publishers, MI, 1980).
80. Curl, C. L., Meierotto, L., Castellano, R. L. S., Spivak, M. R. & Kannan, K. Measurement of urinary pesticide biomarkers among Latina farmworkers in southwestern Idaho. *J. Expo. Sci. Environ. Epidemiol.* **31**, 538–548 (2021).
81. The International Agency for Research on Cancer (IARC). *WHO Classification of Tumours of the Digestive System*. vol. 3 (World Health Organization, 2010).
82. Kleiner, D. E. *et al.* Design and validation of a histological scoring system for nonalcoholic fatty liver disease. *Hepatology* **41**, 1313–1321 (2005).
83. Neuschwander-Tetri, B. A. & Caldwell, S. H. Nonalcoholic steatohepatitis: summary of an AASLD Single Topic Conference. *Hepatology* **37**, 1202–1219 (2003).
84. Scheuer, P. J. Classification of chronic viral hepatitis: a need for reassessment. *J. Hepatol.* **13**, 372–374 (1991).
85. Bressac, B., Kew, M., Wands, J. & Ozturk, M. Selective G to T mutations of p53 gene in hepatocellular carcinoma from southern Africa. *Nature* **350**, 429–431 (1991).
86. Laurent-Puig, P. & Zucman-Rossi, J. Genetics of hepatocellular tumors. *Oncogene* **25**, 3778–3786 (2006).
87. Pineau, P. *et al.* Chromosome instability in human hepatocellular carcinoma depends on p53 status and aflatoxin exposure. *Mutat. Res.* **653**, 6–13 (2008).
88. Mulligan, C. J., Hunley, K., Cole, S. & Long, J. C. Population genetics, history, and health patterns in Native Americans. *Annu. Rev. Genomics Hum. Genet.* **5**, 295–315 (2004).
89. Lott, M. T. *et al.* mtDNA variation and analysis using Mitomap and Mitomaster. *Curr. Protoc. Bioinformatics* **44**, 1.23.1-26 (2013).
90. Maggi, F., Tang, F. H. M., la Cecilia, D. & McBratney, A. PEST-CHEMGRIDS, global gridded maps of the top 20 crop-specific pesticide application rates from 2015 to 2025. *Sci. Data* **6**, 170 (2019).
91. Đikić, D. *et al.* Carbendazim combined with imazalil or cypermethrin potentiate DNA damage in hepatocytes of mice. *Hum. Exp. Toxicol.* **31**, 492–505 (2012).
