# Supplementary material for: Mapping pesticide mixtures to cancer risk at the country scale with spatial exposomics
Source: Nat Health. 2026 Apr 1;1(5):520–31. doi: 10.1038/s44360-026-00087-0 (PMC13156043; doi:10.1038/s44360-026-00087-0)
Supplement: Supplementary file 2 — Reporting Summary [file 44360_2026_87_MOESM2_ESM.pdf]

nature portfolio

Corresponding author(s): Stéphane Bertani

Last updated by author(s): Oct 31, 2025

Reporting Summary

Nature Portfolio wishes to improve the reproducibility of the work that we publish. This form provides structure for consistency and transparency in reporting. For further information on Nature Portfolio policies, see our Editorial Policies and the Editorial Policy Checklist.

Statistics

For all statistical analyses, confirm that the following items are present in the figure legend, table legend, main text, or Methods section.

n/a	Confirmed	
☐	☒	The exact sample size (*n*) for each experimental group/condition, given as a discrete number and unit of measurement
☒	☐	A statement on whether measurements were taken from distinct samples or whether the same sample was measured repeatedly
☐	☒	The statistical test(s) used AND whether they are one- or two-sided<br>*Only common tests should be described solely by name; describe more complex techniques in the Methods section.*
☐	☒	A description of all covariates tested
☐	☒	A description of any assumptions or corrections, such as tests of normality and adjustment for multiple comparisons
☐	☒	A full description of the statistical parameters including central tendency (e.g. means) or other basic estimates (e.g. regression coefficient) AND variation (e.g. standard deviation) or associated estimates of uncertainty (e.g. confidence intervals)
☐	☒	For null hypothesis testing, the test statistic (e.g. *F*, *t*, *r*) with confidence intervals, effect sizes, degrees of freedom and *P* value noted<br>*Give P values as exact values whenever suitable.*
☐	☒	For Bayesian analysis, information on the choice of priors and Markov chain Monte Carlo settings
☐	☒	For hierarchical and complex designs, identification of the appropriate level for tests and full reporting of outcomes
☒	☐	Estimates of effect sizes (e.g. Cohen's *d*, Pearson's *r*), indicating how they were calculated

Our web collection on statistics for biologists contains articles on many of the points above.

Software and code

Policy information about availability of computer code

Data collection

All open-source datasets used in this study, including version details and access information, are provided in Supplementary Table 2. Additionally, KoboToolbox (v2.020.25) and OpenRefine (v3.4.1) were used for data collection and data cleaning, respectively.

Data analysis

The custom R scripts for generating the process-based pesticide exposure risk map and for integrated nested Laplace approximation (INLA) modelling are available in the Figshare repository at https://doi.org/10.6084/m9.figshare.29728463. Access credentials for a test version of the code are available from the corresponding author upon reasonable request. All other analyses relied on available software, fully referenced in the manuscript, including:
- Bioconductor (v.3.20),
- Excel (v.16.16.27),
- Fred's Softwares,
- GraphPad Prism (v.10.4.1),
- Mitomaster (v.Beta 1),
- Nominatim (v.4.5.0),
- PostgreSQL (v.16.3),
- Python (v.3.11.3),
- QGIS (v.3.28 LTR), and
- QuantaSoft (v.1.7)
- R (v.4.4.1), R packages included: dplyr (v.1.1.4), gghalves (v.0.1.4), ggplot2 (v.3.5.2), ggrain (v.1.0.2), INLA (v.25.3.24), Plotly (v.4.10.4), raincloudplots (v.0.2.0), sf (v.1.0-10), sp (v.1.5-1), SpatialEpi (v.1.2.8), spdep (v.1.3-10), sva (v.3.54.0), terra (v.1.8-70), and tmap (v.4.0).

For manuscripts utilizing custom algorithms or software that are central to the research but not yet described in published literature, software must be made available to editors and reviewers. We strongly encourage code deposition in a community repository (e.g. GitHub). See the Nature Portfolio guidelines for submitting code & software for further information.

Data

Policy information about availability of data

All manuscripts must include a data availability statement. This statement should provide the following information, where applicable:

- Accession codes, unique identifiers, or web links for publicly available datasets
- A description of any restrictions on data availability
- For clinical datasets or third party data, please ensure that the statement adheres to our policy

The datasets generated and analysed during the current study are available in the Figshare repository at https://doi.org/10.6084/m9.figshare.29728463, except for individual-level geolocation data from GEO and the National Cancer Institute of Peru (INEN) Cancer Registry, which are not publicly available to protect privacy and comply with data protection regulations. Transcriptomic data analysed are publicly available in GEO under accession numbers GSE111580 and GSE136247 (Peru). Comparative datasets were obtained from GEO under accession numbers GSE17548 (Turkey), GSE45436 (Taiwan), and GSE62232 (France). No new individual-level data were collected, and informed consent was not required.

Research involving human participants, their data, or biological material

Policy information about studies with human participants or human data. See also policy information about sex, gender (identity/presentation), and sexual orientation and race, ethnicity and racism.

Reporting on sex and gender	Sex was recorded from medical records and corresponds to biological sex assigned at diagnosis; gender identity was not documented. Although the primary objective focused on spatial patterns of tumour incidence and environmental exposures irrespective of sex or gender, stratification by sex (female, male) and age group (0–39, 40–59, ≥60 years) was applied when calculating standardized incidence ratios (SIRs). No further sex- or gender-based stratification was conducted in the geospatial or transcriptomic analyses, and all findings apply to both sexes. Sex-disaggregated clinical epidemiological data for the Peruvian liver cancer cohort are available; individual-level data are included only where participant consent and ethics approval permitted data sharing.
Reporting on race, ethnicity, or other socially relevant groupings	Ethnicity, race, or other socially defined categories were not used as variables in the study design or analyses. Such groupings were not incorporated in geospatial or transcriptomic analyses, and no adjustments for them were performed. However, in the Peruvian liver cancer cohort, matrilineal ancestry data were included to provide context on genetic background and population structure. These were inferred by researchers through mitochondrial haplogrouping, based on the established classification of Native American haplotypes (A–D) from reference sequences. These data were not interpreted as proxies for social constructs.
Population characteristics	Covariate-relevant population characteristics included age, sex, residential address at diagnosis, and primary cancer diagnosis (ICD-10 codes C00–C96), obtained from the INEN cancer registry. These variables informed stratification of cancer incidence ratios and geospatial modelling. Additionally, clinical records of 36 Peruvian liver cancer patients were reviewed to extract covariates relevant to aetiology and progression, including hepatitis B status, history of alcohol use or metabolic disorders (e.g. steatosis or diabetes), and any treatment administered.
Recruitment	Participants were not actively recruited; rather, the study retrospectively included all eligible cases recorded in the INEN cancer registry from 2007 to 2020. INEN holds the most comprehensive oncology dataset in the country, comprising cases diagnosed and treated at the national referral centre. Potential sources of bias include referral and diagnostic imbalances due to the under-representation of remote or underserved regions with limited access to specialist care, and possible under-ascertainment of early-stage or non-histologically confirmed cancers.
Ethics oversight	The Cancer Registry at INEN is authorised by the National Authority for the Protection of Personal Data (ANPD) under registration number RNPDP-EP 4794. The study also received approval from the INEN Institutional Review Board under reference numbers 113-2014-CIE/INEN, 407-2016-CIE/INEN, and 049-2024-DICON/INEN.

Note that full information on the approval of the study protocol must also be provided in the manuscript.

Field-specific reporting

Please select the one below that is the best fit for your research. If you are not sure, read the appropriate sections before making your selection.

☒ Life sciences ☐ Behavioural & social sciences ☐ Ecological, evolutionary & environmental sciences

For a reference copy of the document with all sections, see nature.com/documents/nr-reporting-summary-flat.pdf

Life sciences study design

All studies must disclose on these points even when the disclosure is negative.

Sample size	No sample-size calculation was performed. Instead, we included all eligible cancer cases recorded in the INEN registry from 2007 to 2020. This period was selected to capture trends consistent with chronic environmental exposures, including pesticides, and to align broadly with the modelled pesticide risk window (2014–2019). After applying exclusion criteria to ensure data quality, the final dataset comprised 158,072 primary cancer cases (C00–C96), offering robust statistical power for spatial and lineage-specific analyses.

Data exclusions	Exclusion criteria were pre-established to ensure data integrity and geographic precision. Recurrent cases, duplicates, and internally inconsistent records were removed. To minimise misclassification of chronic exposure, individuals without verified residency at the recorded address for at least five years prior to diagnosis were excluded. These criteria ensured the inclusion of primary cancer cases with robust diagnostic and spatial data suitable for geostatistical analyses.
Replication	This study relies on retrospective analysis of existing registry and environmental data; no experimental procedures were performed that require replication. All inclusion criteria and analytical methods are fully documented to enable reproducibility using comparable datasets.
Randomization	This study did not involve random allocation, as all cancer cases were retrospectively analysed. Tumours were assigned post hoc to biologically coherent groups based on the developmental lineage classification of neoplasms introduced by Jules J. Berman. To reconcile biological relevance with statistical robustness, tumours were stratified at the fifth hierarchical level of this taxonomy. Spatial models accounted for potential confounding through structured and unstructured random effects.
Blinding	Blinding was not relevant to this study, as no experimental interventions or subjective outcome assessments were involved.

Reporting for specific materials, systems and methods

We require information from authors about some types of materials, experimental systems and methods used in many studies. Here, indicate whether each material, system or method listed is relevant to your study. If you are not sure if a list item applies to your research, read the appropriate section before selecting a response.

Materials & experimental systems

n/a	Involved in the study
☒	☐ Antibodies
☒	☐ Eukaryotic cell lines
☒	☐ Palaeontology and archaeology
☒	☐ Animals and other organisms
☐	☒ Clinical data
☒	☐ Dual use research of concern
☒	☐ Plants

Methods

n/a	Involved in the study
☒	☐ ChIP-seq
☒	☐ Flow cytometry
☒	☐ MRI-based neuroimaging

Clinical data

Policy information about clinical studies

All manuscripts should comply with the ICMJE guidelines for publication of clinical research and a completed CONSORT checklist must be included with all submissions.

Clinical trial registration	Not applicable.
Study protocol	Not applicable.
Data collection	Data were collected from the cancer registry maintained by INEN in Lima (Peru), covering the period 2007–2020.
Outcomes	Not applicable.

Plants

Seed stocks	*Report on the source of all seed stocks or other plant material used. If applicable, state the seed stock centre and catalogue number. If plant specimens were collected from the field, describe the collection location, date and sampling procedures.*
Novel plant genotypes	*Describe the methods by which all novel plant genotypes were produced. This includes those generated by transgenic approaches, gene editing, chemical/radiation-based mutagenesis and hybridization. For transgenic lines, describe the transformation method, the number of independent lines analyzed and the generation upon which experiments were performed. For gene-edited lines, describe the editor used, the endogenous sequence targeted for editing, the targeting guide RNA sequence (if applicable) and how the editor was applied.*
Authentication	*Describe any authentication procedures for each seed stock used or novel genotype generated. Describe any experiments used to assess the effect of a mutation and, where applicable, how potential secondary effects (e.g. second site T-DNA insertions, mosiacism, off-target gene editing) were examined.*
